# Performance of Dental Students in Understanding and Retention of Oral Pathology Concepts: A Comparative Analysis of Traditional versus Live-Field Teaching Methods

**DOI:** 10.1155/2022/3257377

**Published:** 2022-07-04

**Authors:** Anitha Salian, Swathi Sanal Kumar, Srikant Natarajan, Amitha J. Lewis, Nidhi Manaktala, Dilip G Naik, Karen Boaz, Nandita KP, Shweta Yellapurkar

**Affiliations:** ^1^Department of Oral Pathology and Microbiology, Manipal College of Dental Sciences, Mangalore, Manipal Academy of Higher Education, Manipal, India; ^2^Department of Periodontics, Manipal College of Dental Sciences,, Mangalore, Manipal Academy of Higher Education, Manipal, India

## Abstract

**Background:**

Understanding oral aspects of pathology by traditional techniques has always been a paradigm in the field of dental education. Traditional methods of teaching include interaction using black board, projectors, and alternate methods of teaching such as a student-centered approach where live-field demonstrations, audio visual aids, and student interaction are also gaining importance, ultimately promoting active education. The aim of the study was to compare live-field and static-field teaching methods in understanding and retention of the histopathological features in dental students.

**Methods:**

This was a cross-sectional analytical study, wherein a uniform cohort of III-year dental students was obtained by randomizing the study subjects. Practical classes were conducted using traditional black board/static pictures and dynamic live-field teaching comprising of microscope connected to an HD screen and projector demonstrating the preferred microscopic field. Alternately, the level of retention of knowledge was measured using customized topic-based tests. The comparison of average scores was done between live-field and static-field teaching groups using the paired *t*-test.

**Results:**

The test scores using the paired *t*-test were marginally elevated in the conventional mode of teaching; however, it varied with respect to precise topics taken using both the genres of teaching.

**Conclusion:**

A balance of both conventional and virtual teaching needs to be achieved to enhance the comprehension in student learning. Nevertheless, in the impending years, advanced research is entailed to see if the virtual mode of teaching could replace the conventional method for the advancement in the study prospects.

## 1. Background

For a building to last decades without breaking apart, the maximum importance is given to its foundation. Similarly, education is the strong foundation which is the prerequisite for nurturing the nation's present and future generations. At present, our educational society is divided into two categories, the group which supports and promotes the use of modern technologies like computers, laptops, tablets, smart phones for educational purpose inside and outside the classroom and the group which still supports traditional methods like black board or white board in classroom. The traditional methods of teaching include teacher-centered method of interaction (teacher to student) using black board, projectors and pictures. On the other hand, the alternate method of teaching is a student-centered approach where live-field demonstrations, audio visual aids, and student interaction are more emphasized [1]. The dilemma about usage of a particular method alone is that not every student benefits from a single method. Despite its advantages, virtual learning has few technical concerns and student isolation is a major concern. Some students prefer the interaction in a regular classroom, whereas self-directed learners prefer online education [2]. Modern teaching aids can offer valid solutions to some teaching and learning problems associated with shortage of manpower and other resources, providing course material in an affordable, electronic format and more interactive which has minimum restrictions on time and space [3]. In medical/dental field of education, integrated learning can be most effective. Integrated education can provide the theory knowledge as well as give a practical approach side by side, and it can encourage the learner to have a better understanding of the subject [4]. The understanding of histology, embryology, or pathology starts with the interpretation of the morphology of cells, tissues, and organs and later pathogenesis. However, until recently, the access to visual information was limited to periodic and short practical microscopy sessions backed up by textbooks and printed atlases. In the 1980s, video microscopy revolutionized histology classes by remarkably improving the image quality [5, 6]. The integration of live-field interactive demonstrations is a useful component which aids in understanding of the microscopic cellular and architectural features. The use of microscopy, live-field teaching, and recorded lecture videos allows students to incorporate and promote active learning [7]. This new teaching method is slowly finding place within traditional modes of teaching histology, such as classroom lectures, laboratories using light microscopes, and textbooks or histological atlases. Thus, the main aim of the study was to compare the traditional versus live-field teaching methods in understanding and retention of knowledge of histopathological features taught to dental students.

## 2. Methods

*Ethical Statement.* The Institutional Ethics Committee approval has been taken prior to the commencement of the study ref. no 17106. Informed consent was waived as the students participated in the examination as a scheduled part of their routine assessment postteaching of a particular topic.

*Study Design.* This was a cross-sectional analytical study. This article was described according to the STROBE (Strengthening and Reporting of Observational Studies in Epidemiology) statement available from https://www.equator-network.org/reporting-guidelines/strobe/. This study was conducted over a period of 3 months.

*Materials and/or Subjects.* The third-year dental students were the study subjects. There was no sampling procedure followed as the third-year dental students enrolled for that particular year (*n* = 98) were in total included for the evaluation. A particular histopathological topic was selected for evaluation, and a faculty member was assigned for the theory and practical class. Theory classes were conducted for the entire batch of students for the designated topics. To have a uniform cohort of students according to the grade point averages (GPA), the students were randomized into two groups (group I and group II) as those having similar median scores in the sessional examination.

Groups I and II had a statistically insignificant difference in GPA. For the practical session, the students were divided as batches A and B based on their roll numbers with both the batches having students from groups I and II randomly. Practical classes were conducted for batch A based on the traditional method and batch B based on the live-field method.

The traditional method was lecture-based that used static pictures of histopathology slides, power point presentation, and a black/white board. The live-field histopathological method utilized a live-field projection of the histopathology slides using a microscope (Olympus CX21I) connected to a camera and the field being focused was displayed on a high-definition television (Samsung smart display signage DB48e) screen and LCD (NEC Asia Pacific) projectors.

**Figure 1:**
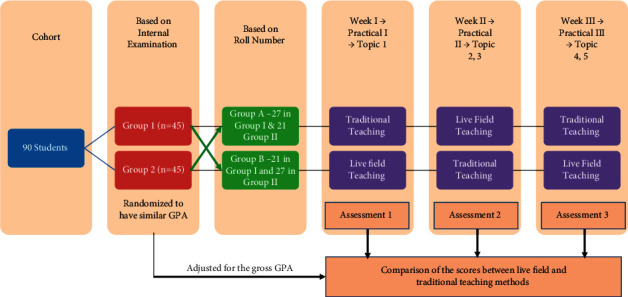
The sequence of randomization and classes uptaken for the score assessment.

Following the completion of live-field and traditional method of teaching for the whole batch of students, the next consecutive theory class was utilized for assessment. The questions were structured in such a way as to assess the understanding of the cell morphology in terms of size, shape, and epithelial connective tissue interface objectively. The data collected (grades) were compared to identify the level of cognition (knowledge) of each topic covered using the two methods. To avoid bias, classes were conducted by the same staff member and none of the students were previously aware of the topics covered. Topics were completed for both groups of students in the equivalent time frame. Both groups had the same content comprising of the similar teaching methods alternatively. The sequence was repeated thrice and 5 topics were covered as per the flowchart mentioned (Figure 1). The questionnaire consisted of set of questions based on tumors and cysts with the choice of multiple options. Content validation was done by four experts who have an experience of teaching oral pathology for >5 years. Four questions each on cellular features and on tissue patterning and two questions on pathogenesis were included.

### 2.1. Statistics

The student scores were anonymized and randomized into two groups of live-field and static-field teaching methods. The comparison of average scores was done between live-field and static-field teaching groups using the paired *t*-test. The analysis was done individually for each topic as well as in total.

## 3. Results

There were 26 students in both groups for topic 1, 43 for live-field and 38 for static-field for topics 2 and 3, and 38 for live-field and 43 for static-field for topics 4 and 5. There were no dropouts in the teaching schedules.

Live-field and static-field teaching in pathology had similar performances as per the analysis of the scores of students analyzed using the paired *t*-test. Overall, the static-field teaching had a marginally better score of 5.31 as compared to the live-field score of 5.08 (*P* value of 0.032) (Table 1).

**Figure 2:**
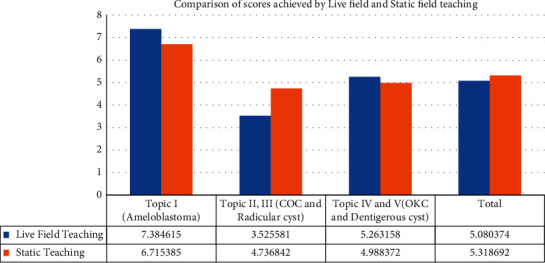
Comparison of scores achieved by students trained with live-field and static-field techniques.

Comparison of the individual topics, however, had variable results. Topics (1, 4, and 5) had better scores in the live-field teaching, while (2 and 3) were significantly better in the static-field teaching (*p* < 0.001) (Figure 2).

## 4. Discussion

Learning tools and technology enable students to develop effective self-directed learning skills. In recent times, medical and dental specialties have benefitted from the use of digital microscopy in the fields of diagnosis, research, and teaching. Traditional and live-field teaching are both learning techniques followed in the field of dentistry with both having pros and cons [8]. A number of studies have given the students' level of perception of these teaching techniques, which directly compare their ability to learn under various formats. However, some studies have also reported that teacher-student interaction is an essential criterion for student's retention of academic knowledge and success [9–11].

As per the analysis of present study scores after the lecture series, the students had equally rated both the teaching formats. The static-field technique allows students to get better associated with the learning material, whereas live-field learning offers a more thought-provoking and involving way to digest information. However, analyzing the scores of students in relation to the individual topics derived a two-way outcome, which shows that topics such as ameloblastoma, odontogenic keratocyst, and dentigerous cyst had better scores in live-field teaching in contrast to the topics such as calcifying odontogenic cyst and radicular cyst which had better scores with static-field teaching.

Sinn et al., reported that activating digital microscopic techniques in teaching modalities facilitated the students' access to the slides beyond the microscopy lab and made it an expedient tool for teaching. In such learning methods an individual's abilities of questioning, solving and feedback may not be possible. However, Isabelle et al., stated that both the teaching formats have its own advantages and disadvantages as there could be lack of reproducibility due to operator bias, need of high-end microscope in the labs, as well as the need for improved skills to operate the digital sources. It promotes direct visualization of all the diagnostic fields with storing and grading of the slides if required. However, the price can range from tens to hundreds of thousands of a currency [12]. Traditional microscopic techniques are practiced worldwide in studying cellular biology and its characteristic features. Its ease of use and affordability has made it an invaluable tool in teaching. The easy handling of the equipment and not requiring any microscopic light or focus adjustments were the major advantages of the static technique with respect to teachers. In addition to this, when the high-quality images were projected onto the screen, the students could relate the pathology more closely to the theoretical aspect of the same [13, 14].

The attitude of the students towards these learning techniques was biased. They reciprocated quite well to live-field as well as static-field techniques, which was evident in their test scores. When approached for the general feedback response, it was certain that traditional techniques are time consuming as they required fields of choice to view the structures of interest, and more often students due to their absence were unable to focus the appropriate field in their consecutive classes on their own. In contrast to the above, through digital microscopy, unlimited number of students were able to view the microscopic images at the same time on the screen, which also conserved time and enabled them to have increased interactive sessions with the faculty with respect to the topics of interest [15]. Specific areas of diagnostic relevance could also be marked on the slide image for better retention of knowledge, and the images could be easily stored for the future academic purposes as they do not deteriorate with time.

Digital microscopy is more precise, due to its image quality and higher resolution. It is digitalized; hence, the images could be stored in various resolutions (4x, 10x, and 40x) and can be accessed at any time by an individual for image analysis [8]. Live-field is more interactive, and specific cellular features and orientation of tissue components can be seen and understood better in this teaching modality. Fields of diagnostic importance could be easily labelled for students' convenience and eludes any operator bias [6, 16]. Topics of ameloblastoma, odontogenic keratocyst, and dentigerous cyst have unique cellular features that was better understood in our study group, using live-field teaching, which emphasizes this fact.

The following were the benefits of inducing live-field techniques with conventional ones:

1. Live-field microscopy enhanced teacher-student interaction sessions and promoted increased level of competency in cognitive domain
2. Live-field video demonstration ensured visualization of cell shape, tissue orientation, and spatial morphology more closely and simultaneously by allowing students interaction
3. Along with the theory, simultaneous teaching of slides was made possible to a large number of students at a time
4. Teachers could manage their time effectively imparting more knowledge and skills to the students and reducing the number of teaching hours. One-on-one interaction could be earmarked for advanced discussion as live-field has shown all the features simultaneously to all the students
5. The discussions were made easier as the slides could be easily annotated and fields of diagnostic relevance were studied better

Fred Dee in his study indicated the ideology behind the implementation of virtual microscopy at the college set-up as it depends on the faculty commitments and also towards these educational programs and their computer-assisted abilities [6]. Karamizadeh et al. showed that integrated learning was efficiently capable of creating docility regarding time, place, and leap of learning, which also indicated that there is significant corelationship between their accessibility and their attitude towards the integrated learning approach [4]. Kristin K et al. in their research stated that the live-field teaching technique helped them to invest their time effectively as it increased teacher-student rapport. The students' queries revolved around the pathological topics rather than technical concern with respect to the microscopic adjustments. He found that an integrated approach of live-field and traditional techniques facilitated additional learning and teaching and also helped to restructure a student community [13]. Inspite of all the benefits of the live-field teaching, static-field represents most diagnostic focus as decided by the pathologist after complete survey of the histopathological slide. Under live-field examination, the diverse presentation of the histopathological features of the lesion becomes evident to the students. This may blur the concept and diagnostic features required to identify the lesion in the present study. Additionally, the quality of slide in terms of staining, the variability in histopathological features shown in slide, the attitude and concentration of the students to learning at a given point of time are the limitations of the present study.

## 5. Conclusion

Pathology does not read textbooks; thus, the pathognomic diagnostic field may not always be visualized or present in the histopathology slide. Static-field identifies this pathognomic field for teaching, but in reality, the students should understand the variations are better taught by live-field teaching. Far-flung research is entailed to see if integrated learning alone could overcome the contemporary study curriculum, by overlooking its limitations. The current study inferred that to teach practical histology/pathology, computer-generated education may be an effective adjunct to the conservative method.

## Figures and Tables

**Table 1:** The paired *t*-test for comparison of the scores achieved by live-field and static-field teaching.

Topic	Teaching	*N*	Mean	Standard deviation	Standard error mean	*t*	*P* value
Topic 1	Live-field teaching	26	7.384615	1.240546	0.243291	1.908	0.062
	Static-field teaching	26	6.715385	1.287693	0.252537		

Topics 2 and 3	Live-field teaching	43	3.525581	1.245773	0.189979	−4.333	**<0.001**
	Static-field teaching	38	4.736842	1.266709	0.205487		

Topics 4 and 5	Live-field teaching	38	5.263158	1.369242	0.22212	0.954	0.343
	Static-field teaching	43	4.988372	1.222256	0.186392		

Overall	Live-field teaching	107	5.080374	1.98173	0.191581	−0.997	0.32
	Static-field teaching	107	5.318692	1.478917	0.142972		

## Data Availability

The data used to support the findings of this study are available from the corresponding author upon request.
